# Elevated C-reactive protein to albumin ratio is a promising predictive biomarker for prognosis in patients with renal cell carcinoma

**DOI:** 10.1080/07853890.2026.2618431

**Published:** 2026-01-21

**Authors:** Chunhua Xu, Yifan Guo, Xiaoni Chen, Xi Liu, Fengfang Wu, Shan Lin

**Affiliations:** ^a^ Department of Nephrology, Longgang Central Hospital of Shenzhen, Shenzhen, China; ^b^ Shenzhen Hospital, Beijing University of Chinese Medicine, Shenzhen, China

**Keywords:** C-reactive protein to albumin ratio, renal cell carcinoma, prognostic value, inflammation

## Abstract

**Background and aims:**

Systemic inflammation is closely linked to the development and progression of various cancers, as well as poorer patient outcomes. The role of the c-reactive protein to albumin ratio (CAR) as a predictor of poor prognosis in renal cell carcinoma (RCC) remains insufficiently recognized.

**Methods:**

A literature search was conducted in major English-language databases, including PubMed, EMBASE, and the Cochrane Library, with the search updated to 25 June 2025. Both the odds ratio (OR) and diagnostic odds ratio (DOR) were employed to evaluate the prognostic performance of CAR.

**Results:**

This meta-analysis ultimately included 10 studies with a total of 2478 patients with RCC. The results showed that high baseline CAR was significantly associated with poor prognosis or recurrence of RCC. The sensitivity of 0.73 (95% confidence interval [CI]. 0.69–0.77); specificity of 0.69 (95% CI: 0.64–0.74) and DOR of 6.0 (95% CI: 5.0–8.0) were pooled estimated from patient-based analyses. Subsequently, the combined positive likelihood ratio (PLR) and negative likelihood ratio (NLR) were calculated with the results of 2.4 (95% CI: 2.0–2.8) and 0.39 (95% CI: 0.33–0.46), respectively. Furthermore, the area under the curve (AUC) of the summary receiver operating characteristic (SROC), which reflects prognostic accuracy, was 0.77 (95% CI: 0.73–0.81). In addition, subgroup analysis indicated that elevated CAR was more predictive of overall survival (OS) in RCC patients in China.

**Conclusions:**

Our findings indicate that an elevated CAR serves as a strong predictor of poor prognosis or recurrence in patients with RCC.

## Introduction

Renal cell carcinoma (RCC) accounts for approximately 2–3% of all malignant neoplasms worldwide. Among its various subtypes, clear cell renal cell carcinoma (ccRCC) is the most prevalent histopathological subtype, constituting approximately 70–80% of all RCC cases [1]. As a highly aggressive and frequently lethal malignancy of the urinary system, RCC has been associated with a continuously rising incidence, increasing at an approximate annual rate of 2% [2]. Notably, this upward trend may be partially attributable to the recent recognition of favorable subsets of cancers of unknown primary (CUP), most prominently the RCC-like CUP subtype, which is managed according to RCC treatment paradigms and thus may contribute to observed incidence patterns [3]. This upward trend has established RCC as a significant global public health challenge. Despite ongoing advancements in multimodal therapeutic strategies, RCC remains a leading cause of cancer-related mortality [4], largely due to its high propensity for metastasis and recurrence following surgical intervention, which significantly compromises long-term patient outcomes.

Traditionally, the prognostic evaluation of RCC has relied primarily on established pathological parameters, such as the tumor-node-metastasis (TNM) staging system and Fuhrman nuclear grading [5]. However, these conventional indicators exhibit significant limitations in accurately predicting individual patient outcomes. Specifically, their ability to discriminate between prognostic categories is suboptimal in patients with intermediate risk, resulting in uncertainties in clinical decision-making [6]. Therefore, there is a critical need for robust, reproducible, and clinically applicable biomarker that enable early detection, precise diagnosis, and reliable prognostic stratification in RCC. The development and validation of such biomarkers would play a pivotal role in advancing personalized therapeutic strategies and improving long-term survival outcomes for patients with this malignancy. Notably, recent advances in medical diagnostic technology have facilitated earlier detection of RCC and improved prognostic accuracy; among these, exosomes have emerged as a promising source of non-invasive tumor biomarkers. Due to their small size, high mobility, and unique bilayer membrane structure, exosomes can readily cross biological barriers while protecting encapsulated bioactive molecule from degradation by extracellular RNases and proteases. This protective capacity enhances the stability of these molecular cargoes, making exosomes highly sensitive tools for disease detection [7]. Importantly, miRNA species derived from tumor-associated exosomes have been detected in the serum and urine of RCC patients, offering non-invasive targets for early diagnosis, disease monitoring, and prediction of tumor behavior, including invasion and metastatic potential [7].

Systemic inflammatory responses have been well documented to play critical roles in the initiation, progression, and unfavorable prognoses of a wide spectrum of malignant neoplasms [8,9]. Within the RCC tumor microenvironment, inflammatory cells and their secreted cytokines are actively involved not only in tumor development but also in disease progression through multiple mechanisms, including the promoting of angiogenesis and the suppressing anti-tumor immune responses [10]. ccRCC as the most common RCC subtype, is driven by loss of functional von Hippel-Lindau (VHL) protein, leading to stable hypoxia-inducible factor 2α (HIF-2α) and sustained activation of HIF signaling [11]. Belzutifan, a selective HIF-2α inhibitor, blocks its dimerization with HIF-1β, preventing transcriptional activation, and suppresses tumor growth. In a phase III trial, belzutifan outperformed everolimus in previously treated advanced ccRCC, with higher 18-month progression-free survival (24.0% vs. 8.3%) and objective response rate (21.9% vs. 3.5%) [11]. Against this background, various inflammation-related biomarkers have been proposed. Among them, C-reactive protein (CRP), a widely recognized acute-phase protein, has demonstrated considerable utility in prognostic evaluation across multiple cancer types [12]. Concurrently, albumin serves as a key indicator of nutritional status and liver function, with reduced levels being closely associated with poorer clinical outcomes in cancer patients [13]. However, both biomarkers have specific limitations. CRP concentrations can be elevated by numerous non-malignant conditions, leading to relatively low diagnostic specificity. Conversely, albumin levels are influenced by multiple physiological variables, such as nutritional status and hepatic function, which may compromise its sensitivity as a standalone prognostic marker. Therefore, the C-reactive protein to albumin ratio (CAR), a novel composite score reflecting systemic inflammation, has been emerged as a promising biomarker and has garnered increasing scholarly attention in recent years [14].

The CAR simultaneously reflects the systemic pro-inflammatory status mediated by CRP and the anti-inflammatory or nutritional status indicated by albumin. This dual representation enables a more comprehensive assessment of both systemic inflammation and nutritional imbalance. Moreover, the ratio-based calculation may amplify minor fluctuations in individual biomarkers, thereby enhancing sensitivity to pathological changes [15]. Additionally, as a continuous variable, CAR preserves a higher degree of original data integrity, contributing to improved accuracy in prognostic prediction. Accumulating evidence from studies on various malignancies, such as hepatocellular carcinoma and esophageal cancer, indicates that CAR may outperform individual biomarkers (e.g. CRP or albumin alone) as well as established composite scores like the Glasgow Prognostic Score (GPS) and its modified version (mGPS) in prognostic stratification [16,17]. In the context of RCC, although research on CAR is still in its early stages, emerging studies have suggested its potential utility in prognostic evaluation [18]. Nevertheless, most existing studies are limited by small sample sizes and heterogeneous methodologies, leading to inconsistent findings. Therefore, a systematic evaluation of the current clinical evidence is urgently needed to clarify elucidate the prognostic role of CAR in RCC and to assess its potential for clinical implementation.

In this meta-analysis, we aim to summarize the current body of evidence regarding CAR, with a focus on its prognostic significance, potential mechanistic underpinnings, and future clinical applicability. It is anticipated that this analysis will provide a robust evidence base and valuable insights to guide further research and clinical decision-making. In addition, we conducted several subgroup analyses to assess differences in predictive performance across countries or regions and according to methodological or and dimensional divisions.

## Materials and methods

### Data collection and search strategies

A systematic search was conducted across multiple English-language databases, including PubMed, EMBASE, and the Cochrane Library, covering the period from their inception to 25 June 2025. The following search terms were used: (‘renal cell carcinoma’ [MeSH] OR ‘renal carcinoma’ [MeSH] OR ‘renal cancer’ [MeSH] OR ‘renal tumor’ [MeSH] OR ‘renal neoplasms’ [MeSH] OR ‘kidney carcinoma’ [MeSH] OR ‘kidney cancer’ [MeSH] OR ‘kidney tumor’ [MeSH] OR ‘kidney neoplasms’ [MeSH] OR ‘nephron carcinoma’ [MeSH] OR ‘nephron tumor’ [MeSH] OR ‘nephron neoplasms’ [MeSH] OR ‘RCC’ [MeSH]), combined with (‘inflammatory markers’ [Title/Abstract], OR ‘c-reactive protein to albumin ratio’ [Title/Abstract], OR ‘c-reactive protein-to-albumin ratio’ [Title/Abstract], OR ‘c-reactive protein albumin ratio’ [Title/Abstract], OR ‘CRP/Alb ratio’ [Title/Abstract], OR ‘CAR’ [Title/Abstract]). Complete search results and a summary of the retrieval outcomes were incorporated into this study. Additionally, the reference lists of retrieved articles were manually screened to identify any potentially relevant studies not captured by the electronic search. The meta-analysis was conducted in accordance with the PRISMA guidelines (Preferred Reporting Items for Systematic Reviews and Meta-Analyses) [19,20].

### Selection of studies

A systematic and sequential screening process was applied to all retrieved references. Full-text articles of potentially relevant studies were obtained based on their titles and abstracts. Two independent reviewers (Chunhua Xu and Yifan Guo) assessed each study for eligibility. Any disagreements regarding inclusion were resolved through discussion with a third reviewer (Shan Lin), who served as a mediator. Studies were included if they met the following criteria: (1) serum CAR levels were measured before the initiation of treatment; (2) all participants were adults aged 18 years or older; (3) the sample size exceeded 20 individuals; (4) the study design was either observational or a randomized controlled trial (RCT); and (5) the study provided sufficient data or allowed for the derivation of false positive (FP), true positive (TP), false negative (FN), and true negative (TN) values to evaluate the predictive accuracy of CAR in patients with RCC. Studies were excluded if any of the following conditions applied: (1) insufficient reporting of prognostic performance data for either the control or intervention group; (2) the study was limited to animal or *in vitro* models; (3) the data were duplicated or inadequately described; or (4) the publication type was classified as a review, conference abstract, commentary, editorial, or supplementary material.

### Data extraction

The experimental data were independently extracted by Chunhua Xu and Xiaoni Chen. Any discrepancies between the two reviewers were resolved *via* discussion with a third independent reviewer (Xi Liu), who facilitated consensus through adjudication. For each study included in the analysis, prespecified data elements were systematically collected, including the following parameters: first author’s name, geographic origin (country or region), year of publication, study design, enrollment period, total sample size (with gender distribution when available), median age (in years), area under the curve (AUC) with 95% confidence interval [CI], diagnostic sensitivity, diagnostic specificity, and the baseline CAR cutoff value.

### Assessment of study quality

In the present study, the Cochrane Risk of Bias Tool was independently applied by two reviewers (Chunhua Xu and Yifan Guo) to assess the methodological rigor of the included studies [21]. The tool assesses six key domains: random sequence generation, allocation concealment, blinding of participants and personnel, blinding of outcome assessment, incomplete outcome data, selective reporting, and other potential sources of bias. Each domain was judged as ‘low risk’, ‘high risk’, or ‘unclear risk’ of bias, with individual items rated as ‘yes’, ‘no’, or ‘uncertain’.

Additionally, the Newcastle-Ottawa Scale (NOS) was used to evaluate the quality of the included observational studies [22]. This scale evaluates three core aspects: study population selection, comparability of groups, and ascertainment of exposure or outcome, using a star-based scoring system with a maximum of nine stars. Studies were classified into three quality levels: scores of 0–3 were considered low quality, 4–6 as moderate quality, and 7–9 as high quality. The implementation of the NOS in this review followed established methods from prior studies, with minor adaptations [23].

### Statistical analysis

All statistical analyses, including the calculation of TN, TP, FP, and FN rates for each individual study, were conducted using Stata version (version 12.0; StataCorp, College Station, TX). The diagnostic performance of the marker was evaluated by estimating pooled sensitivity, specificity, diagnostic odds ratio (DOR), positive likelihood ratio (PLR), and negative likelihood ratio (NLR) [24]. To evaluate inter-study variability, heterogeneity was examined using the Cochran’s Q statistic and *I^2^* test. A *Q* statistic *p*-value < 0.05 or an *I^2^* value greater than 50% was interpreted as indicative of significant heterogeneity. In cases of substantial heterogeneity (*I^2^* > 50%), a random-effects model was applied to pool the data [25]. The Hardy-Weinberg equilibrium (HWE) in the control population of each study was tested using Pearson’s χ^2^ test, with deviations deemed statistically significant at *p* < 0.05 [26].

To further evaluate the predictive capability of CAR in patients with RCC, summary receiver operating characteristic (SROC) curves and forest plots depicting pooled sensitivity and specificity were constructed. The AUC was served as the principal summary indicator [27]. Additionally, subgroup analyses were carried out according to geographic or regional classifications. Finally, potential publication bias was investigated using both Begg’s and Egger’s tests, with results considered statistically significant at *p* < 0.05 [28,29].

## Results

### Included literature

**Figure 1.**
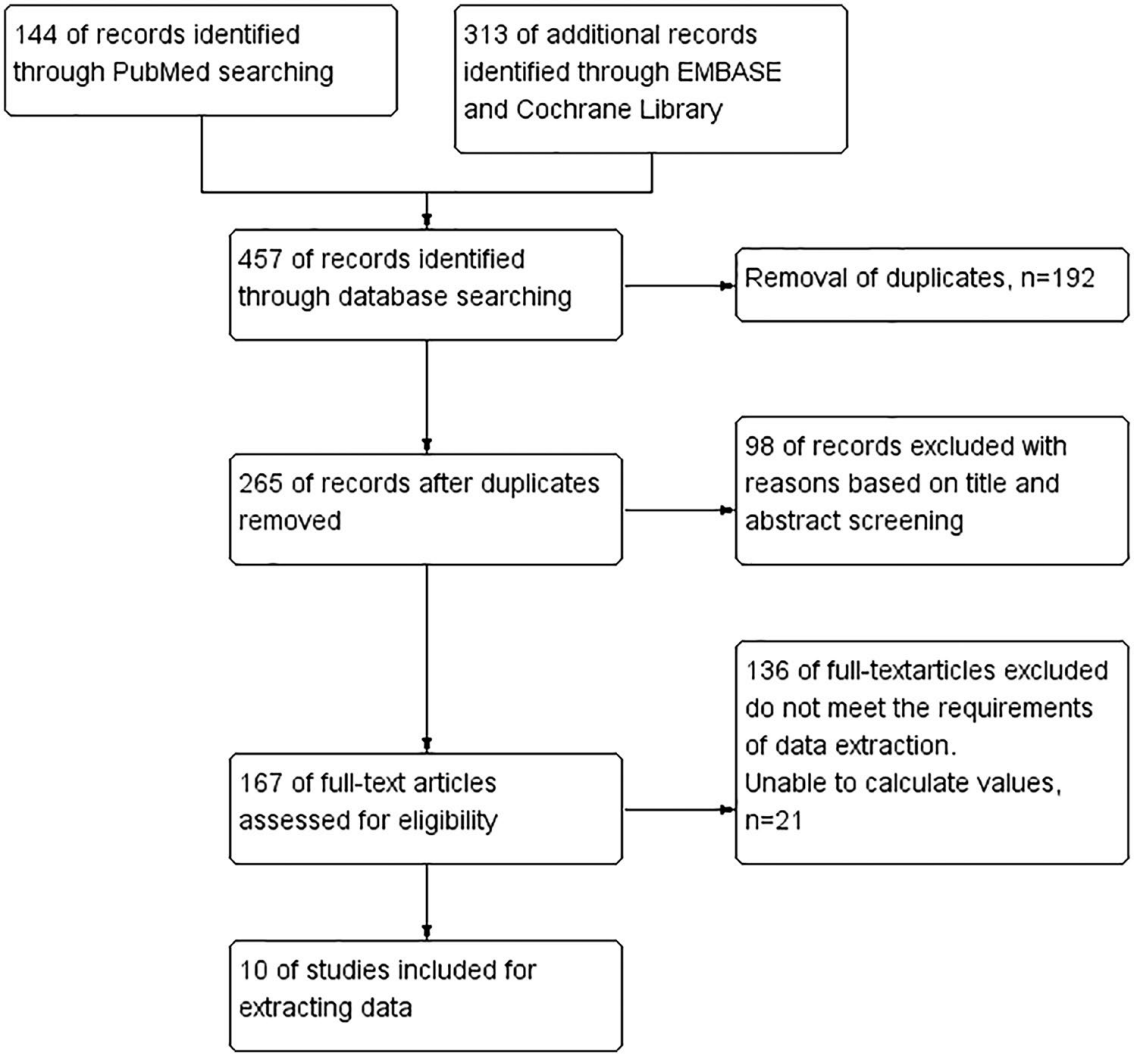
The study selection process for this meta-analysis conducted systematically according to predefined inclusion criteria.

An initial systematic search across electronic databases identified 457 potentially relevant studies. After removing duplicates (*n* = 192), 98 studies were excluded during the initial screening stage based on title and abstract review due to clear irrelevance. A detailed full-text evaluation of the remaining 167 studies led to the exclusion of 136 that did not meet the predefined eligibility criteria, along with an additional 21 studies excluded for specified methodological or reporting-related reasons. Ultimately, 10 rigorously selected high-quality studies were incorporated into the meta-analysis. These studies collectively enrolled 2478 patients and were utilized to comprehensively assess the prognostic relevance of CAR in patients diagnosed with RCC. The systematic, multi-phase study selection process is summarized in Figure 1.

### The characteristics and quality of the included studies

All ten studies were published in the English language. To assess the methodological quality of the included studies, baseline characteristics were systematically extracted and are summarized in Table 1. Of the 10 trials, 8 were in East Asia and the remaining 2 was in South Asia or West Asia, including 5 in mainland China, 3 in Japan. All studies are from single-center clinical trials between 2015 and 2024. All the 10 observational studies included 2478 RCC patients, including 1272 in mainland China [30,31,32,33,34], 1088 in Japan [35,36,37], 31 in India [38], and 87 in Turkey [39]. Table 2 summarizes the predictive power of CAR in the prognosis of patients with RCC. The AUC is between 0.630 and 0.820, and the cut-off value is between 0.025 ng/mL and 0.11 ng/mL. In addition, sensitivities and specificities were calculated or given for the included tests in the ranges 0.667 to 0.875 and 0.430 to 0.790, respectively.

**Table 1.** Characteristics of the studies included in this meta-analysis.

Author (Year of publication)	Country	Study Design	Sample size (male)	Age (years)	Treatment	Survival endpoints	Survival analysis	Follow-up (months) Median (range)	NOS
Agizamhan (2018)	China	RTP, MI	82 (33)	37 (2–71) R	Surgery	OS/DFS/CSS	Multivariate Univariate	31 (2–108)	9
Barua (2019)	India	RTP, SC	31 (21)	62 ± 3.14	Surgery	OS/PFS	Multivariate	16.5	7
Chen (2015)	China	RTP, SC	406 (253)	58 (24–80) R	Surgery	OS	Multivariate Univariate	63 (1–151)	8
Gao (2019)	China	RTP, SC	108 (78)	57 (23–78)R	Surgery	OS/DFS	Multivariate Univariate	54.5 (7.3–74.2)	9
Guo (2017)	China	RTP, SC	570 (382)	51.4 ± 13.5	Surgery	OS/DFS/CSS	Multivariate Univariate	63.54	9
Konishi (2019)	Japan	RTP, MI	176 (129)	67 (59–74)	Targeted therapy	OS/PFS	Multivariate	NR	7
Liu (2024)	China	RTP, SC	106 (76)	57.8 (13.167)	Surgery	OS	Multivariate Univariate	NR	7
Makino (2023)	Japan	RTP, MI	213 (155)	63 (18–85) R	Surgery	OS/DFS/CSS/PFS	Multivariate Univariate	53.4 (0.36–157.32)	8
Tsujino (2019)	Japan	RTP, MI	699 (500)	61.9 ± 11.7	Surgery	OS/CSS	Multivariate Univariate	73	8
Uzun (2022)	Turkey	RTP, MI	87 (68)	63 (21–81) R	NR	OS/DFS/PFS	Multivariate	NR	8

*Note*. NOS: Newcastle-Ottawa Scale; RTP: retrospective; SC: single center; MI: multi-institutional; NR: not reported; OS: overall survival; DFS: disease-free survival; PFS: progression-free survival; CSS: cancer-specific survival; R: range.

**Table 2.** The predictive value of CAR for poor prognosis of RCC.

Study	AUC	*p* Value of AUC	Cut-off value(ng/mL)	Sensitivity(%)	Specificity(%)	Number of patients			
						TP FP FN TN			
Agizamhan (2018)	0.772	0.003	0.083	70.6	72.8	31	10	13	28
Barua (2019)	0.750	0.021	0.11	78.0	43.0	11	10	3	7
Chen (2015)	0.790	<0.001	0.060	70.0	79.0	170	34	73	129
Gao (2019)	0.820	<0.001	0.094	80.0	77.3	29	16	7	56
Guo (2017)	0.715	<0.001	0.080	66.7	75.1	262	44	131	133
Konishi (2019)	0.720	<0.05	0.05	70.0	68.0	78	20	34	44
Liu (2024)	0.811	0.004	0.0452	87.5	69.5	7	29	1	66
Makino (2023)	0.714	0.0074	0.025	71.4	60.8	68	46	27	72
Tsujino (2019)	0.820	0.029	0.073	80.9	70.8	202	131	48	318
Uzun (2022)	0.630	0.047	0.072	72.0	54.0	45	29	18	34

### Assessment of methodological quality and publication bias

**Figure 2.**
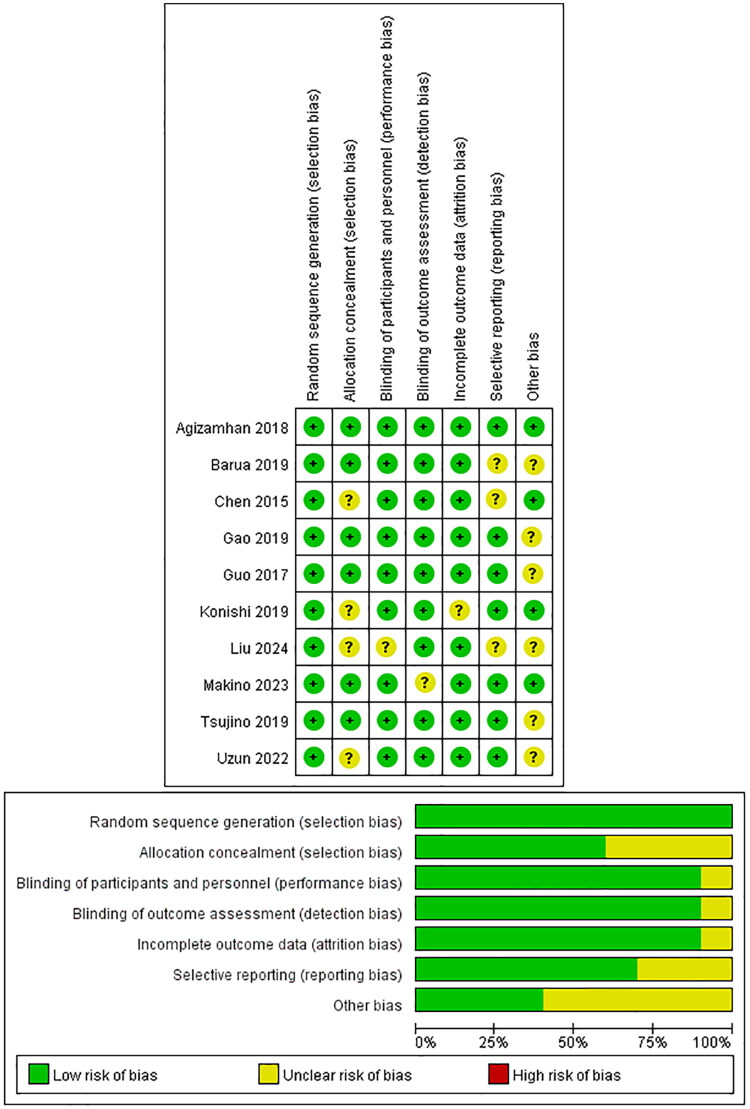
The quality assessment of all included eligible studies systematically conducted using the Cochrane Risk of Bias Assessment Tool. This rigorous evaluation process contributed to the robustness of the meta-analysis by identifying studies with low, unclear, or high risk of bias, thereby enhancing the validity of the synthesized findings.

This study ensured that all included trials specified clear inclusion criteria and excluded predefined participant populations. The methodological quality of each selected study, all of which achieved a Cochrane score of 10 or higher, was systematically assessed using The Cochrane Risk of Bias Tool. Additionally, the overall methodological quality of the included studies was rated as moderate. The results of the Cochrane risk of bias assessment are presented in Figure 2.

**Figure 3.**
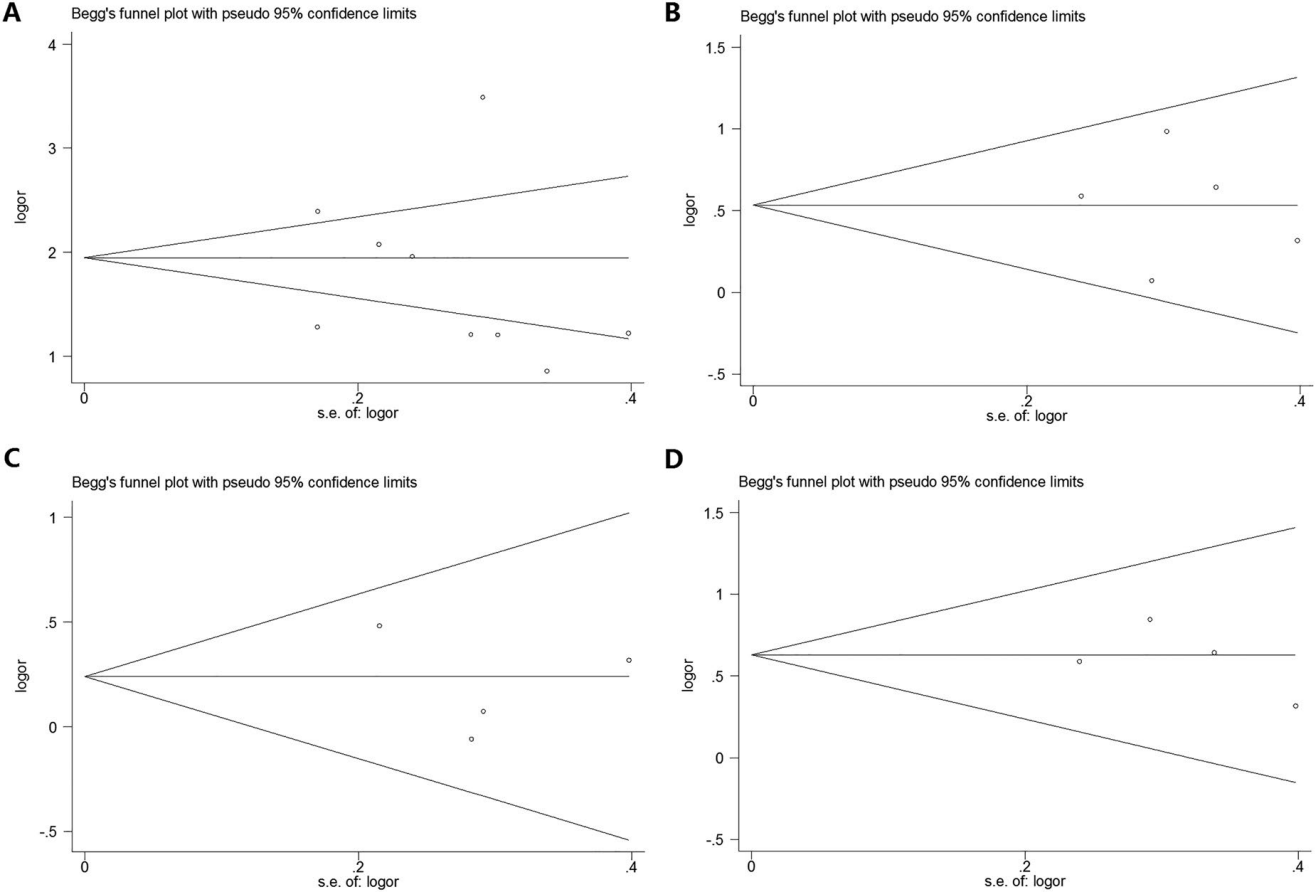
The Begg’s funnel plot employed as a graphical method to assess the presence of publication bias in the included studies. (A) Begg’s test for OS. (B) Begg’s test for DFS. (C) Begg’s test for PFS. (D) Begg’s test for CSS.

To further assess the credibility of the meta-analysis results, a Begg’s funnel plot was used as a graphical method to examine potential publication bias among the selected studies examining the prognostic value of CAR. The symmetry observed in the funnel plots indicated no significant asymmetry with respect to key survival outcomes, including overall survival (OS), disease-free survival (DFS), progression-free survival (PFS), and cancer-specific survival (CSS) (Figure 3). Subsequently, Egger’s regression test was conducted to provide a more rigorous quantitative assessment, with statistical set at *p* < 0.05. The results showed no statistically significant publication bias across the studies for OS (*p* = 0.942), DFS (*p* = 0.509), PFS (*p* = 0.815), and CSS (*p* = 0.514). These visual analyses suggest a relatively balanced distribution of the included studies, without noticeable gaps or clusters that might indicate selective publication or reporting bias. Collectively, these findings support the robustness of CAR as a stable and reliable prognostic biomarker across the evaluated research.

### CAR for predicting prognosis in patients with RCC

**Figure 4.**
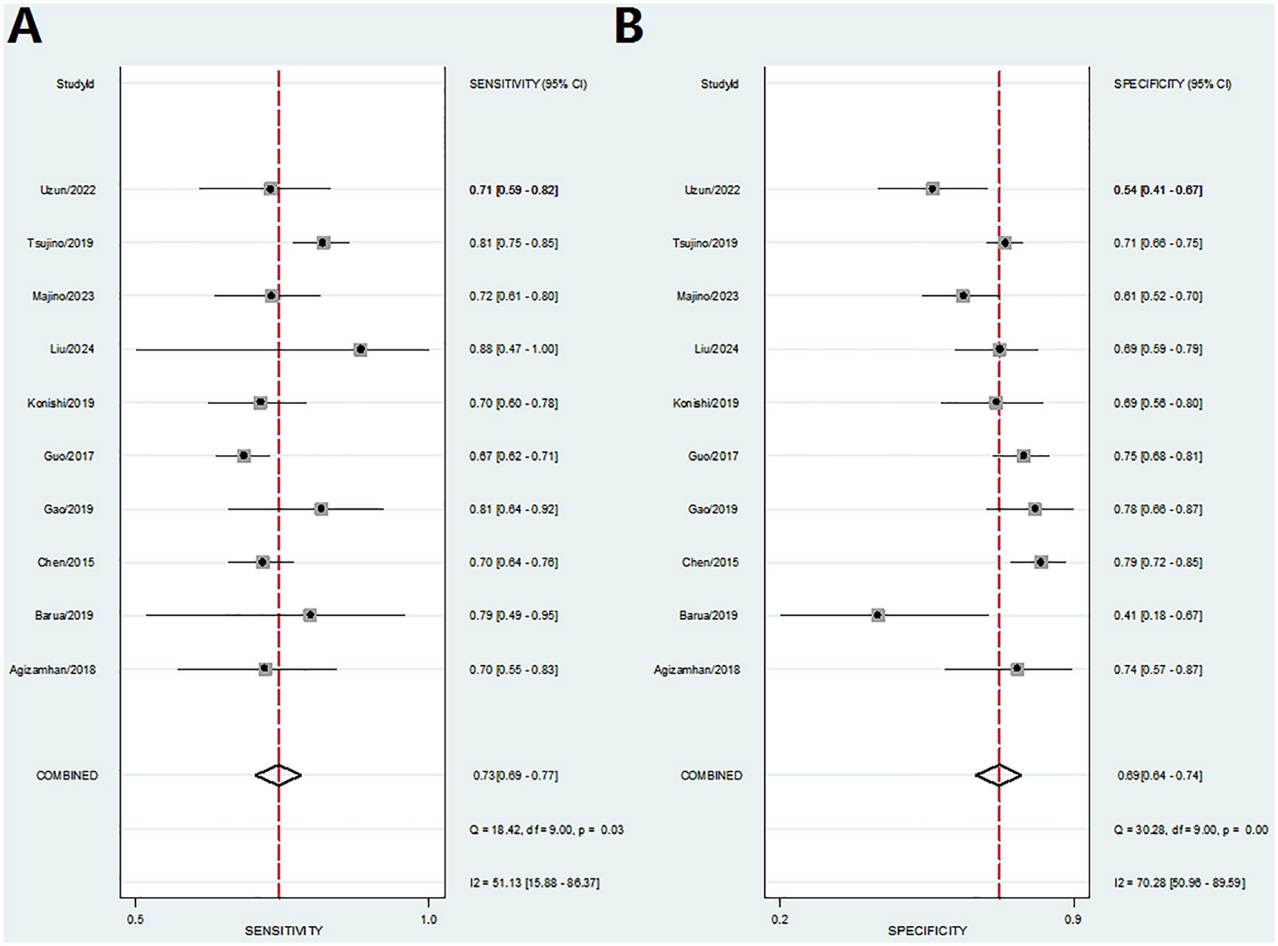
Forest plot illustrating the sensitivity and specificity of elevated CAR in predicting RCC prognosis. (A) Sensitivity. (B) Specificity.

A total of ten datasets were extracted from ten eligible studies (Table 2), including key parameters such as the AUC, 95% confidence interval (CI), optimal cutoff value of CAR, sensitivity, specificity, as well as TP, FP, FN, and TN values. These ten studies collectively evaluated the prognostic potential of CAR as a biomarker in patients with RCC, involving a total of 2,478 individuals. The aggregated data are summarized in Table 2. In assessing the diagnostic performance of CAR, the pooled sensitivity was 0.73 (95% CI: 0.69–0.77) (Figure 4(A)), with a pooled specificity of 0.69 (95% CI: 0.64–0.74) (Figure 4(B)). Additionally, the PLR was 2.4 (95% CI: 2.0–2.8), and the NLR was 0.39 (95% CI: 0.33–0.46). Furthermore, the pooled DOR was 6.85 (95% CI: 5.68–8.27) calculated using a random-effects model.

**Figure 5.**
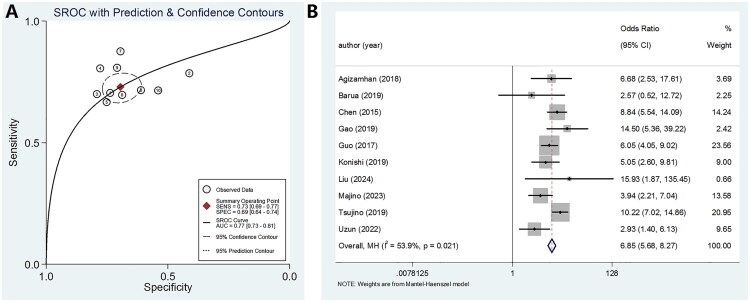
Performance evaluation of CAR in predicting the prognosis of patients with RCC. (A) Forest plot of AUC of high CAR predicting RCC prognosis. (B) Forest plot of the predictive value of high CAR on the prognosis of RCC patients.

Additionally, a forest plot was constructed to visually display the AUC values associated with elevated CAR levels in predicting the prognosis of RCC. This graphical representation, shown in Figure 5(A), summarizes the diagnostic performance across all included studies, offering a comprehensive overview of CAR’s predictive capability. To assess heterogeneity, the shape of the SROC curve was examined. Notably, no distinct ‘shoulder-arm’ pattern was observed within the SROC space, which strongly indicates the absence of a threshold effect—suggesting that variations in CAR cut-off values across studies did not substantially influence the overall diagnostic performance. The area under the SROC curve, which serves as a global measure of prognostic accuracy, was estimated to be 0.77 (95% confidence interval: 0.73–0.81), indicating that CAR demonstrates moderate but consistent discriminatory ability in predicting clinical outcomes for RCC patients. To further illustrate the prognostic value of CAR, the association between CAR levels and patient survival outcomes is summarized in Figure 5(B), which highlights the trend toward poorer prognosis in patients with higher CAR values at diagnosis. Taken together, these findings support the conclusion that an elevated CAR level at the time of diagnosis or treatment initiation is significantly associated with less favorable clinical outcomes in individuals with RCC, underscoring its potential utility as a prognostic biomarker in clinical practice.

### Subgroup analysis

To explore potential variations in the predictive value of elevated CAR levels for recurrence and adverse prognosis in RCC patients across different regional variations, a comprehensive subgroup analysis was performed, with the results summarized” in Table 3. Notable disparities were observed in the prognostic performance of CAR when comparing DOR and AUC values across populations. Regional subgroup analysis revealed that both the DOR and AUC of CAR were higher in studies conducted in China than in those from non-Chinese regions (DOR: 7 vs. 5; AUC: 0.79 vs. 0.74). These results indicate that the association between elevated CAR levels and the risk of recurrence or poor prognosis appears to be stronger compared to those from other geographical regions.

**Table 3.** Subgroup analysis of prognostic value of CAR for patients with RCC.

Studies	No. of studies	No. of patients	Sensitivity	Specificity	PLR	NLR	DOR	AUC
Geographical region								
China	5	1272	0.69(0.65–0.72)	0.76(0.72–0.79)	2.8(2.4–3.3)	0.41(0.37–0.46)	7(5–9)	0.79(0.75–0.82)
Non-China	5	1206	0.74(0.68–0.79)	0.63(0.56–0.70)	2.0(1.6–2.5)	0.42(0.31–0.56)	5(3–8)	0.74(0.70–0.78)
Sample size								
≥200	4	1888	0.73(0.67–0.78)	0.72(0.66–0.78)	2.6(2.2–3.2)	0.38(0.31–0.46)	7(5–10)	0.79(0.75–0.82)
Cut-off value (ng/mL)								
≤0.06	4	901	0.71(0.66–0.75)	0.70(0.63–0.77)	2.4(1.9–3.0)	0.42(0.35–0.50)	6(4–8)	0.73(0.68–0.76)
>0.06	6	1577	0.74(0.68–0.80)	0.68(0.60–0.76)	2.4(1.8–3.1)	0.38(0.29–0.48)	6(4–10)	0.78(0.74–0.81)
Treatment								
Surgery	8	2215	0.74(0.69–0.78)	0.71(0.66–0.76)	2.6(2.2–3.0)	0.37(0.31–0.44)	7(5–10)	0.79(0.75–0.82)
Survival endpoints								
OS	10	2478	0.73(0.69–0.77)	0.69(0.64–0.74)	2.4(2.0–2.8)	0.39(0.33–0.46)	6(5–8)	0.77(0.73–0.81)
DFS	5	1060	0.70(0.65–0.74)	0.69(0.60–0.76)	2.2(1.7–2.8)	0.44(0.38–0.52)	5(3–7)	0.72(0.68–0.76)
PFS	4	507	0.71(0.66–0.76)	0.60(0.54–0.66)	1.8(1.5–2.1)	0.48(0.39–0.59)	4(3–5)	0.71(0.66–0.74)
CSS	4	1564	0.73(0.66–0.79)	0.70(0.65–0.75)	2.5(2.1–3.0)	0.38(0.30–0.48)	6(4–9)	0.77(0.73–0.80)
Survival analysis								
Multivariate	10	2478	0.73(0.69–0.77)	0.69(0.64–0.74)	2.4(2.0–2.8)	0.39(0.33–0.46)	6(5–8)	0.77(0.73–0.81)
Univariate	8	2184	0.74(0.69–0.78)	0.71(0.66–0.76)	2.6(2.2–3.0)	0.37(0.31–0.44)	7(5–10)	0.79(0.75–0.82)

In the survival endpoint subgroup analysis, the prognostic value of CAR in predicting RCC recurrence and adverse outcomes was more pronounced in patients assessed for OS, compared to those evaluated for DFS (DOR: 6 vs. 5; AUC: 0.77 vs. 0.72), and PFS (DOR: 6 vs. 4; AUC: 0.77 vs. 0.71), while showing comparable performance for CSS (DOR: 6 vs. 6; AUC: 0.77 vs. 0.77). When stratified by the CAR cut-off value (≤0.06 vs. >0.06), the prognostic accuracy remained similar between the two subgroups (DOR: 6 vs. 6; AUC: 0.73 vs. 0.78). In the subgroup analysis according to the type of survival analysis, the univariate analysis group demonstrated slightly higher than DOR and AUC values compared to the multivariate analysis group (DOR: 7 vs. 6; AUC: 0.79 vs. 0.77), indicating a potentially greater predictive effect when not adjusting for multiple confounding factors.

### Analysis of sensitivity and heterogeneity

A sensitivity analysis was performed to assess the robustness of the pooled results for OS and disease-free/progression-free/cancer-specific survival (DFS/PFS/CSS). In this process, each individual study was sequentially omitted to examine the influence of any single dataset on the combined OR. The pooled OR estimates for both outcome categories remained stable unchanged after the removal of any single study (results not shown), indicating that no single study disproportionately exerted a disproportionate impact on the overall findings. These results demonstrate that the meta-analytic estimates are robust and dependable.

Additionally, potential sources of heterogeneity were explored using meta-regression and subgroup analyses (Table 3). A CAR cut-off value greater than 0.06 ng/mL (*I^2^* = 62.4%, *p* = 0.021) and studies conducted outside China (*I^2^* = 72.7%, *p* ≤ 0.005) were identified as significant contributors to the observed heterogeneity. In contrast, other factors examined did not significantly account for the heterogeneity in the prognostic performance of CAR. For subgroups exhibiting substantial heterogeneity, a random-effects model was applied to pool effect sizes. Conversely, in subgroups where heterogeneity was absent, a fixed-effects model was utilized for combining effect estimates.

### The relation of CAR with clinicopathological features of RCC

**Figure 6.**
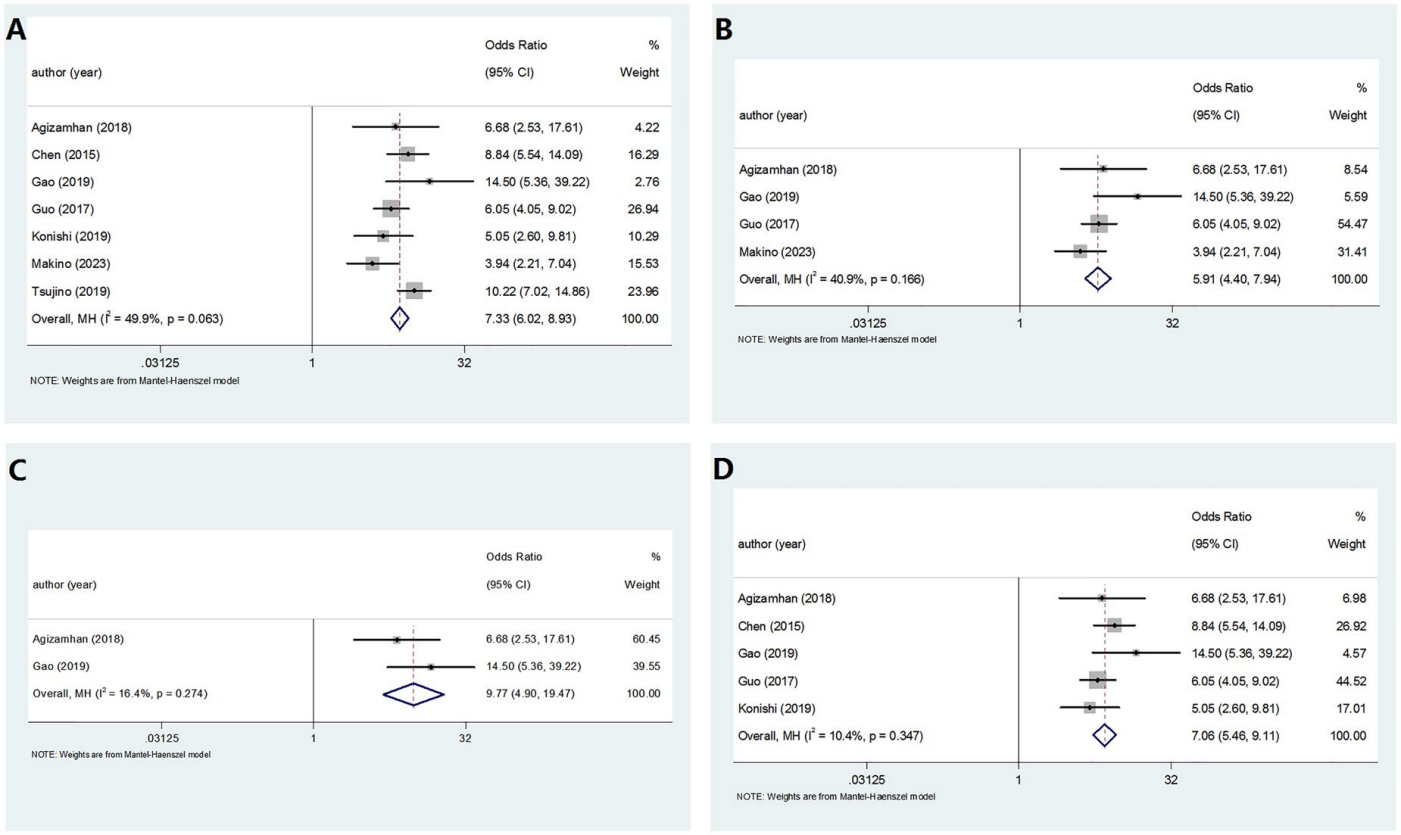
Meta-analyses of the association between CAR and clinicopathological parameters in patients with RCC. (A) Gender (male vs. female). (B) Fuhrman grade (grades 1–2 vs. grades 3–4). (C) Tumor laterality (left vs. right). (D) TNM stage (stages I–II vs. stages III–IV).

A total of seven studies involving 2,254 patients investigated the association between CAR and clinicopathological features in RCC. Pooled analyses revealed no significant links between CAR levels and key clinical parameters. Specifically, no significant association was found between CAR and patient gender (OR = 7.33, 95% CI = 6.02–8.93, *p* = 0.063), suggesting that the prognostic significance of CAR is consistent across both male and female patients. Similarly, no notable correlation was observed between CAR and tumor grade (OR = 5.91, 95% CI = 4.40–7.94, *p* = 0.166), indicating that CAR’s predictive performance is not influenced by the degree of tumor differentiation or histological aggressiveness. Furthermore, no significant link was detected between CAR and tumor laterality (OR = 9.77, 95% CI = 4.90–19.47, *p* = 0.274), implying that the prognostic value of CAR remains stable regardless of tumor site. In addition, the analysis revealed no significant association between CAR and TNM stage (OR = 7.06, 95% CI = 5.46–9.11, *p* = 0.347), demonstrating that CAR’s prognostic relevance is not affected by the anatomical extent of spread as classified by the TNM staging system. These results are summarized in Figure 6 and further detailed in Table 4, providing further insights into the role of systemic inflammatory responses in the progression and prognosis of RCC.

**Table 4.** The correlation between CAR and clinicopathological features in patients with RCC.

Variables	No. of studies	No. of patients	AUC	OR (95%CI)	*p*	Heterogeneity: *I^2^* (%)
Gender (male vs. female)	7	2254	0.79(0.75–0.82)	7.33(6.02–8.93)	0.063	49.9
Fuhrman grade (1 to 2 vs. 3 to 4)	4	973	0.73(0.69–0.77)	5.91(4.40–7.94)	0.166	40.9
Laterality (Left vs. Right)	2	190	NR	9.77(4.90–19.47)	0.274	16.4
TNM stage (I-II vs. III-IV)	5	1342	0.79(0.75–0.82)	7.06(5.46–9.11)	0.347	10.4

## Discussion

Current evidence collectively indicates that the CAR has considerable utility in the prognostic evaluation of RCC. A consistent body of research has demonstrated that elevated preoperative CAR levels are significantly and independently associated with reduced OS and PFS in patients with RCC [40,41]. These findings have been corroborated across multiple studies conducted in diverse geographic regions and involving heterogeneous patient populations with varying sample sizes. A retrospective cohort study diagnosed with ccRCC found that CAR exhibited superior prognostic performance compared to conventional inflammation-based scoring systems, including the GPS and mGPS [37]. Moreover, emerging evidence suggests that CAR not only serves as a valuable prognostic marker in ccRCC but also maintains significant predictive efficacy in less common RCC subtypes, including papillary renal cell carcinoma (pRCC) and chromophobe renal cell carcinoma (chRCC), thereby supporting its broader applicability across histological variants of RCC [41].

Compared to conventional inflammatory prognostic scoring systems such as the GPS and mGPS, the CAR offers several distinct advantages [42]. The GPS and mGPS systems typically categorize CRP and albumin levels into a limited number of discrete groups (usually scored from 0 to 2). This categorical approach inevitably results in the loss of continuous data and underlying biological variability inherent in the original measurements. In contrast, CAR, as a continuous variable, preserves more granular information on systemic inflammation and nutritional status, thereby enhancing the precision of prognostic predictions. A substantial body of clinical evidence has demonstrated that CAR consistently exhibits superior discriminatory ability, as reflected by a higher AUC, in predicting patient survival outcomes—whether analyzed as continuous or dichotomized variable—when compared to GPS and mGPS [43]. Moreover, the calculation of CAR is methodologically simple, requiring only the ratio of CRP to albumin, whereas GPS and mGPS involve separate assessments of each biomarker followed by the integration into a composite scoring system. This computational efficiency makes CAR more feasible for routine clinical application, especially in healthcare settings with limited resources [44]. Importantly, integrating CAR with established clinicopathological parameters, such as TNM stage and Fuhrman nuclear grade, may generate a synergistic effect, further improving the accuracy and clinical utility of prognostic stratification [45].

The clinical utility of the CAR as a prognostic biomarker for RCC is supported by well-established biological mechanisms. Chronic systemic inflammation contributes to tumor progression through multiple complex pathways [46]. Inflammatory cells and their secreted cytokines, such as interleukin-6 (IL-6) and tumor necrosis factor-alpha (TNF-α), can activate key intracellular signaling cascades, including signal transducer and activator of transcription 3 (STAT3) and nuclear factor kappa-light-chain-enhancer of activated B cells (NF-κB) [47]. These activations promote tumor cell proliferation and inhibit apoptosis. Moreover, the inflammatory tumor microenvironment facilitates cancer progression and metastasis through mechanisms such as angiogenesis, epithelial-mesenchymal transition (EMT), and immune evasion. CRP, a sensitive marker of systemic inflammation, is elevated under pro-inflammatory conditions and reflects the activity of a tumor-supportive microenvironment [48]. Notably, down-regulation of lactotransferrin, a critical component of the innate immune system, has been shown to promote metastatic progression in RCC [49]. Importantly, this down-regulation is concurrently associated with increased sensitivity of RCC tumor cells to mTOR inhibitors, suggesting that lactotransferrin may serve as a predictive biomarker for therapeutic response [49]. Conversely, hypoalbuminemia may reflect adverse pathophysiological states, including impaired hepatic synthesis due to systemic inflammation, deteriorating nutritional status, and increased protein catabolism or loss. Albumin performs essential physiological functions, including maintenance of colloid osmotic pressure, scavenging reactive oxygen species as an antioxidant, and transport of various bioactive molecules [50]. Reduced albumin levels may compromise these functions, thereby intensifying oxidative stress, destabilizing the tissue microenvironment, and weakening immune surveillance—collectively creating a favorable environment for tumor growth and dissemination [51]. By integrating both pro-inflammatory and anti-inflammatory/nutritional status into a single composite index, CAR provides a more comprehensive assessment of the ‘inflammation-nutrition imbalance’ axis, which has been recognized as a critical determinant [52].

Despite its promising potential in the context of RCC, the clinical application of the CAR faces several challenges. Current studies investigating the prognostic utility of CAR in RCC are subject to a number of methodological and interpretative limitations. First, most existing studies employ retrospective designs, which are inherently prone to selection bias. Furthermore, considerable heterogeneity exists across studies regarding patient inclusion criteria, methods for determining CAR cutoff values, and follow-up duration. These inconsistencies limit the comparability and generalizability of findings across different study populations. Secondly, the standardization of CAR cutoff values remains a critical challenge. Significant variability in the threshold levels used across studies has been reported, likely due to several factors, including differences in demographic and clinical characteristics of study cohorts (e.g. geographic distribution, disease stage), variations in laboratory measurement techniques (such as conventional versus high-sensitivity CRP assays), and discrepancies in statistical methodologies employed for cutoff derivation. Moreover, although a significant association between CAR and RCC prognosis has been consistently reported, the underlying biological mechanisms remain incompletely understood. Further mechanistic studies are warranted to clarify the precise role of CAR in tumor biology and host systemic responses. In addition, it is necessary to incorporate primary data collection in future research, including the design of a multicenter prospective study to collect clinical data from RCC patients.

Moving forward, there is a pressing need for large-scale, prospective, multicenter investigations. These investigations should aim to standardize research protocols and analytical criteria, systematically explore the pathophysiological mechanisms underlying CAR, and rigorously validate its prognostic performance across diverse clinical settings. This includes evaluating its predictive value across different tumor stages, histological subtypes, and treatment modalities.

## Conclusion

In summary, our pooled analysis indicates that elevated CAR levels are associated with poorer survival outcomes in patients with RCC. These findings highlight the potential of CAR as a valuable prognostic biomarker, providing clinically relevant information for risk stratification and outcome prediction in RCC patients. Nevertheless, further large-scale, well-designed studies are warranted to fully validate the predictive utility of CAR in RCC prognosis.

## Supplementary Material

PRISMA checklist.doc

## Data Availability

All data generated or analyzed during this study are included in this article. Further enquiries can be directed to the corresponding author.
